# Glutathione Modulation in PVY^NTN^ Susceptible and Resistant Potato Plant Interactions

**DOI:** 10.3390/ijms23073797

**Published:** 2022-03-30

**Authors:** Katarzyna Otulak-Kozieł, Edmund Kozieł, Włodzimierz Przewodowski, Katarzyna Ciacka, Agnieszka Przewodowska

**Affiliations:** 1 Department of Botany, Institute of Biology, Faculty of Biology and Biotechnology, Warsaw University of Life Sciences—SGGW, Nowoursynowska Street 159, 02-776 Warsaw, Poland; 2 Laboratory of Potato Gene Resources and Tissue Culture, Bonin Research Center, Plant Breeding and Acclimatization Institute—National Research Institute, 76-009 Bonin, Poland; w.przewodowski@ihar.edu.pl (W.P.); a.przewodowska@ihar.edu.pl (A.P.); 3 Department of Plant Physiology, Institute of Biology, Faculty of Biology and Biotechnology, Warsaw University of Life Sciences—SGGW, Nowoursynowska Street 159, 02-776 Warsaw, Poland; katarzyna_ciacka@sggw.edu.pl

**Keywords:** glutathione S-transferase, plant–virus interaction, ultrastructure, reduced glutathione, resistance, susceptibility, oxidized glutathione

## Abstract

Glutathione is a metabolite that plays an important role in plant response to biotic stress through its ability to remove reactive oxygen species, thereby limiting the degree of potential oxidative damage. It can couple changes in the intracellular redox state to the development, especially the defense responses, of plants. Several studies have focused on measuring glutathione levels in virus infected plants, but have not provided complete information. Therefore, we analyzed, for the first time, the content of glutathione as well as its ultrastructural distribution related to susceptible and hypersensitive potato–*Potato virus Y* NTN (PVY^NTN^) interaction, with an aim of providing new insight into interactive responses to PVY^NTN^ stress. Our findings reported that the inoculation of PVY^NTN^ caused a dynamic increase in the content of glutathione, not only in resistance but also in susceptible reaction, especially at the first steps of plant–virus interaction. Moreover, the increase in hypersensitive response was much more dynamic, and accompanied by a significant reduction in the content of PVY^NTN^. By contrast, in susceptible potato Irys, the content of glutathione decreased between 7 and 21 days after virus inoculation, which led to a significant increase in PVY^NTN^ concentration. Additionally, our findings clearly indicated the steady induction of two selected potato glutathione S-transferase *StGSTF1* and *StGSTF2* genes after PVY^NTN^ inoculation, regardless of the interaction type. However, the relative expression level of *StGSTF1* did not significantly differ between resistant and susceptible plants, whereas the relative expression levels of *StGSTF2* differed between susceptible and resistant reactions. Therefore, we proposed that *StGSTF2* can act as a marker of the type of response to PVY^NTN^. Our observations indicated that glutathione is an important component of signaling as well as the regulatory network in the PVY^NTN^–potato pathosystem. In resistance responses to PVY^NTN^, this metabolite activates plant defenses by reducing potential damage to the host plant cell, causing a reduction in virus concentration, while it can also be involved in the development of PVY^NTN^ elicited symptoms, as well as limiting oxidative stress, leading to systemic infection in susceptible potato plants.

## 1. Introduction

Glutathione, a tripeptide consisting of cysteine, glutamate, and glycine, is a major reservoir of nonprotein reduced sulfur in plants [1]. The bond between the γ-carboxyl group of glutamate and the amino group of cysteine, which differs from the peptide bonds found in proteins, renders stability to this molecule [2,3,4,5]. The most important reactive component of glutathione is the thiol group of cysteine, which is postulated as responsible for the biological and biochemical functions of this tripeptide [6]. Moreover, glutathione functions as a master regulator of intracellular redox homeostasis, as well as one of the redox buffers in most aerobic cells. In free radical reactions, reduced glutathione (GSH) donates hydrogen atoms and produces a thiol radical. Dixon and Edwards [7] stated that a thiol radical is stable, reacts poorly with other hydrogen donors, and can dimerize forming glutathione disulfide (GSSG). The thiol group of GSH can react with a nucleophile and form GS-conjugate with different compounds. Additionally, as underlined by Foyer and Noctor [8], the thiol–disulfide exchange reactions of glutathione mediate the reversible oxidation and reduction of redox sensitive proteins and play an important role in maintaining the redox state. GSH can be oxidized, directly or indirectly, by reactive oxygen species (ROS). GSH can work as a scavenger, preventing the excessive oxidation of cellular environments [9]. It can also react with other thiols to form mixed disulfides. Furthermore, several functions of GSH involve reversible redox reactions. When GSH acts as an antioxidant, it is oxidized to GSSG; however, under stress condition, GSSG is reduced again by glutathione reductase to GSH. As a result, the glutathione pool is mostly reduced [9]. Sabetta et al. [2] reported that glutathione is a multifaced molecule, formed as a product of sulfur metabolism. The authors also stated that glutathione is a mobile molecule, which is systemically transported, as well as a storage form of reduced sulfur, which can be remobilized when needed. Thus, it performs a broad spectrum of functions in plants [9,10,11,12,13,14]. Importantly, GSH acts as a mediator of important cellular processes, such as cell cycle progression and programmed cell death [12].

Due to its ability to directly or indirectly scavenge ROS, GSH is considered as a key metabolite in plant responses to abiotic and biotic stress, which removes ROS and limits the degree of oxidative damage [8]. GSH is not only a weak antioxidant but can also couple changes in the intracellular redox state to the development and defense responses of plants in an ROS dependent manner [15,16]. Wingate et al. [17] found that, in a *Phaseolus vulgaris* suspension cell culture treated with GSH, this compound was involved in local resistance responses, causing the selective induction of the transcription of different defense genes. Since then, it has been postulated that a relationship exists between GSH increase and pathogen resistance [18,19]. On the other hand, unchanged or decreased levels of GSH have been observed in susceptible cell lines [18,20,21,22]. Moreover, changes in the level of glutathione have also been found in plant–virus interactions. In resistant tobacco plants inoculated with *Tobacco mosaic virus* (TMV), an increased level of glutathione was observed in both infected and upper leaves [23]. Furthermore, in cucumber and Styrian oil pumpkin plants systemically infected by *Zucchini yellow mosaic virus* (ZYMV), the ascorbate–glutathione cycle seemed to be of importance in the detoxification of H_2_O_2_ during virus infection [24].

Plant cytosolic glutathione S-transferases (GSTs; EC 2.5.1.18) are essential enzymes involved in multiple and diverse functions, such as detoxification, signaling, redox homeostasis, plant metabolism, growth regulation, and adaptation to biotic and abiotic stress [25]. These enzymes catalyze the conjugation of GSH on several hydrophobic compounds, as well as performing noncatalytic function as transporters [25]. They also act as a signaling marker of infection by various pathogens [2,7]. Plant GSTs are divided into 14 distinct classes, including *tau* (U), *phi* (F), *theta* (T), *zeta* (Z), *lambda* (L), and many others [7,26,27]. Among these, *phi* and *tau* are highly plant specific and the most abundant [28]. Additionally, transcriptomic, deep sequencing investigations revealed that glutathione metabolism, as well as GST expression profile, is regulated by different plant–virus interactions: Geminivirus [29], Tenuivirus [30] or Tobamovirus [31]. Interestingly, 90 GST genes were confirmed in potato and their conserved domains were also identified [32]. Potato GSTs are divided into 10 classes, including *tau* (66 members), *phi* (5 members), *lambda* (5 members), and *theta* and *zeta* (2 members each). Based on high expression in leaves and pathogen induction, we selected the *phi* classes of *StGST* genes located on *Solanum tuberosum* chromosome VI for the analysis of relative expression in potato–PVY^NTN^ interaction.

Several studies have indicated different relationships between glutathione content in plant–pathogen interactions, but complete information is not available yet. Distinct GSTs play an important role in pathogen resistance, and regulating the increase in glutathione is challenging. Therefore, the different regulations in susceptibility and resistance to virus infection of glutathione and *GSTs* allowed us to analyze glutathione status in the *Solanum tuberosum*—Potato virus Y (PVY^NTN^) pathosystem. We aimed to investigate how the modulation of glutathione content and its distribution at an ultrastructural level can influence the interactions between PVY and susceptible and hypersensitive resistance potato. We also analyzed the expression of selected *StGSTFs* genes as well as GST activity by comparing susceptible and hypersensitive interactions. Our results revealed PVY^NTN^ induced glutathione in susceptible as well as resistant responses at the first stages of plant–virus interactions, but the elevation in hypersensitive response was more dynamic and accompanied by a reduction in virus content. On the other hand, the content and activity of GST in susceptible PVY^NTN^–potato Irys interaction increased only until the symptoms appeared, and then significantly decreased. Additionally, significant differences were noticed in the expression of the selected *StGSTF* gene, which indicate that only one of them can act as a marker of PVY^NTN^ inoculation. Significant changes in the ultrastructural distribution of glutathione were also found between susceptible and resistant response.

## 2. Results

### 2.1. Changes in Concentration of PVY^NTN^ in Leaves of Susceptible Irys and Hypersensitive Neptun Potato Plants

**Figure 1:**
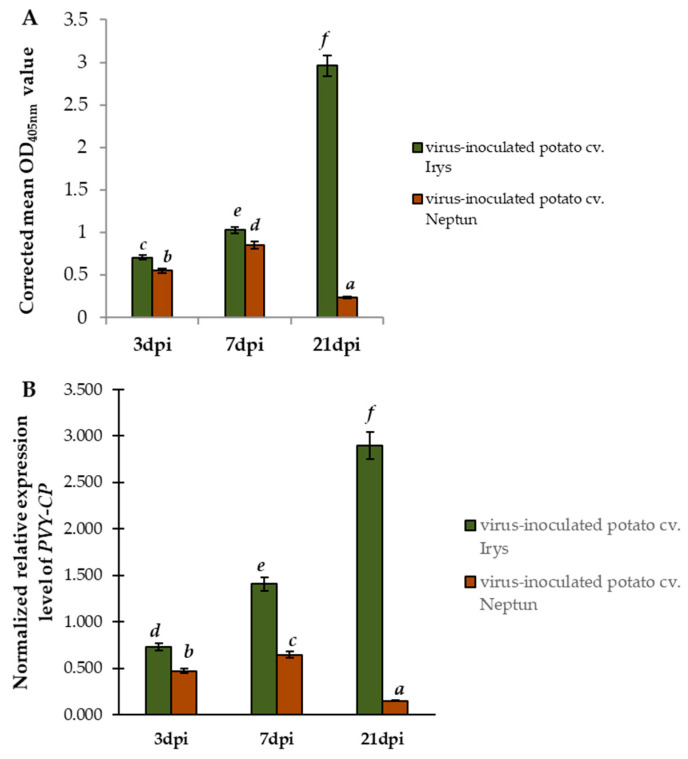
PVY^NTN^ detection and validation of virus concentration in Irys and Neptun plants at 3, 7, and 21 dpi via quantificated DAS-ELISA (**A**) and normalized relative expression of *PVY-CP* (**B**). (**A**) DAS-ELISA detection of PVY. Values represent mean OD_405nm_. (**B**) Normalized relative expression of PVY-CP calculated based on mean expression of *StEf1α* and *Stsec3* reference genes. The statistical significance of differences was assessed at *p* < 0.05 using ANOVA with post hoc Tukey’s HSD (marked by letters above the bars).

Two potato cultivars, Irys and Neptun, were selected to compare susceptible and resistant reactions of the plants to PVY^NTN^, respectively. Neptun is characterized by HR [33,34,35] to PVY [33,34]. DAS-ELISA performed on the samples collected between 3 and 21 dpi confirmed the presence of PVY^NTN^ in both virus inoculated plants. As expected, the virus was not detected in mock inoculated plants (Table S1). The OD_405nm_ values were higher in the Irys plants than in Neptun plants, from 3 to 21 dpi (Table S1). The validation of the corrected mean OD_405nm_ values indicated a statistically significant increase in the relative concentrations of PVY^NTN^ in the Irys cultivar (1.451-fold between 3 and 7 dpi and 2.88-fold between 7 and 21 dpi). On the contrary, in virus inoculated Neptun, the corrected OD_405nm_ mean values showed a significant increase in virus concertation, between 3 and 7 dpi (1.55-fold), and a drastic decrease in concentration, between 7 and 21 dpi (3.58-fold; Figure 1A). To support this observation, the normalized expression of *PVY-CP* was analyzed based on two different plant host reference genes, *StEf1α* and *Stsec3*, to show the virus amount in the inoculated leaves (Figure 1B). The normalized expression of *PVY-CP* changed similarly to the relative PVY concertation based on DAS-ELISA. The *PVY-CP* expression increased in the Irys cultivar (1.92-fold between 3 and 7 dpi and 2.06-fold between 7 and 21 dpi). In the virus inoculated Neptun, *PVY-CP* expression showed a significant increase in virus concertation, between 3 and 7 dpi (1.37-fold), and a drastic downregulation, between 7 and 21 dpi (4.28-fold; Figure 1B). The combined results of the relative PVY concentration and normalized expression of *PVY-CP* suggested that, in Neptun, the resistance reaction began from 7 dpi.

### 2.2. Relative Expression of Selected GST Genes in PVY^NTN^ Differs the Infected Susceptible and Resistance Potato Plants

**Figure 2:**
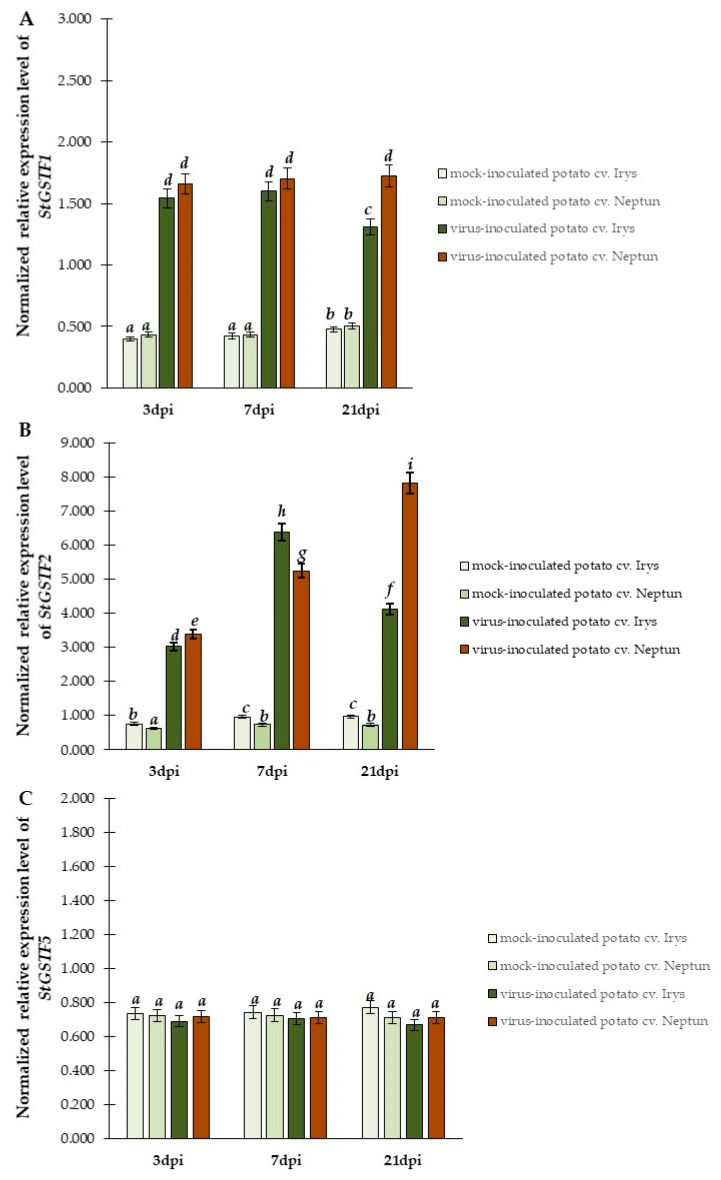
The normalized relative expression levels of *StGSTF1* (**A**), *StGSTF2* (**B**), and *StGSTF5* (**C**) calculated based on mean expression of *StEf1α* and *StSec3* reference genes in mock- and virus-inoculated Irys and Neptun cultivars between 3 and 21 dpi. Using ANOVA and Tukey’s HSD test, the mean values of normalized expression levels were calculated at *p* < 0.05. The statistically significant values are marked by letters above the bar.

The expression of the *StGST* gene is crucial, as it encodes ubiquitous and multifunctional enzymatic protein in the host plant. This enzyme is one of the major phase II detoxification enzymes [36]. In addition to catalyzing the conjugation of electrophilic substrates to reduced glutathione (GSH), these enzymes are involved in a wide range of functions and can noncatalytically bind various endogenous and exogenous ligands. This fact suggests that *StGSTs* could influence the concentration of glutathione and regulate its level, as well as acting as an important factor of resistance or susceptibility to PVY^NTN^. To determine the expression of *StGST1*, *StGSTF2*, and *StGSTF5* in susceptible and resistant plants, qPCRs were performed. The relative expression of *StGSTF1* (Figure 2A) was significantly induced in both cultivars inoculated with PVY^NTN^ compared to mock inoculated plants. However, the level of expression did not significantly differ between resistant and susceptible plants. This may suggest that changes in the expression of *StGSTF1* could not be considered as a marker of reaction type. A different tendency could be observed in the relative expression of *StGSTF2* (Figure 2B) and *StGSTF5* (Figure 2C) in PVY^NTN^ inoculated leaves. The normalized relative expression of *StGST2* indicated steady significant changes from 3 to 21 dpi in virus inoculated plants (both cultivars) **(**Figure 2B). A systematic increase in *StGSTF2* expression was noted in virus inoculated Irys from 3 to 7 dpi (2.11-fold). By contrast, in virus inoculated hypersensitive Neptun plants, the expression of *StGSTF2* increased during 3–21 dpi (2.31-fold). Additionally, mock inoculation did not significantly affect the level of the expression of selected *StGSTF* genes in both cultivars.

In contrast to *StGSTF2,* the changes in the relative expression of *StGSTF5* were not statistically significant. This suggests that, among selected *StGSTF* genes, only the expression of *StGSTF2* differs in resistant and susceptible plants. It also indicates that this gene could be crucial for HR. Moreover, the predicted association of StGSTF2 proteins with chloroplast and cytoplasm [32] implies that StGSTF2 could protect vital plant cell elements during responses to PVY^NTN^. Furthermore, our findings pointed to the relationship between the normalized relative expression of *StGSTF2* in time intervals with relative virus concentration and *PVY-CP* expression. Therefore, we performed an evaluation of the correlation between *StGSTF2* expression and *PVY-CP* expression levels by calculating the PCC separately for susceptible Irys (Table S2A) and hypersensitive Neptun (Table S2B) varieties at 3 dpi, 7 dpi and 21 dpi. The statistical analyses of PCC showed an increase in *StGSTF2* and *PVY-CP* expression in potato Irys plants and confirmed strongly positive correlation between 3 and 7 dpi. Whereas, after 7 dpi, the correlation became negative and a strong decreased expression of *StGSTF2* was correlated with the increase in *PVY-CP* relative expression level (Table S2). On the contrary, at 7 dpi in Neptun plants (when the HR reaction started), the correlation was negative and stronger than in susceptible Irys plants. In hypersensitive Neptun plants, the upregulated expression of *StGSTF2* was corelated with the down regulation of *PVY-CP* (Table S2). This indicates that a correlative high virus concentration and high relative expression level of *StGSTF2* occurred simultaneously, at 7 dpi, in both cultivars. Conversely, between 7 and 21 dpi, i.e., at later stages of responses to PVY^NTN^, the level of *StGSTF2* differed between susceptible and resistant potato plants.

### 2.3. Significant Changes in the Concentration of Reduced (GSH) and Oxidized (GSSG) Forms and GST Activity as Differentiating Factor of Susceptibility and Resistance to PVY^NTN^

**Figure 3:**
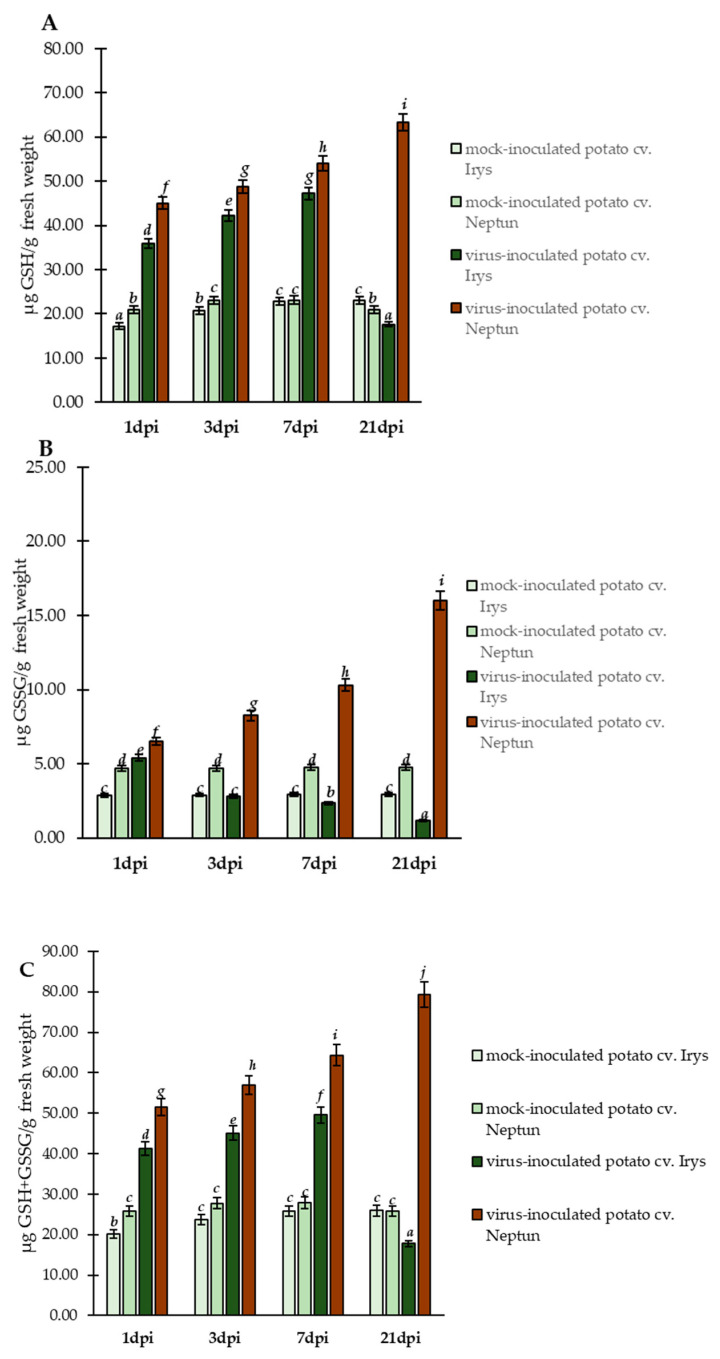
The mean concentration of reduced (GSH) (**A**) and oxidized (GSSG) (**B**) glutathione in the leaves of PVY^NTN^ and mock inoculated susceptible Irys potato and resistance Neptun potato plants between 1 and 21 dpi. (**C**) The mean of summary concentration of reduced (GSH) and oxidized (GSSG) glutathione in the leaves of PVY^NTN^ and mock inoculated Irys and Neptun potato plants between 1 and 21 dpi. Using ANOVA and Tukey’s HSD test, the mean concentrations of GSH and GSSG were calculated at *p* < 0.05. The statistically significant values are marked by letters above the bars.

The determined relative expression levels of selected *StGSTs* highlighted the potential involvement of glutathione itself in the regulation of susceptible or resistant reactions of potato to PVY^NTN^ inoculation. Therefore, HPLC was performed to validate the changes in glutathione forms, GSH and GSSG (Figure 3A,B), and summary glutathione content (Figure 3C) during PVY^NTN^–potato interaction.

**Figure 4:**
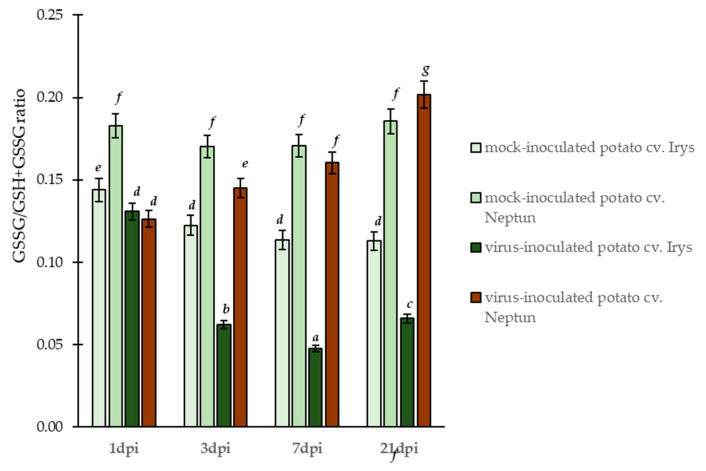
The mean ratio of GSSG/GSH + GSSG concentration in the leaves of PVY^NTN^ and mock inoculated Irys and Neptun potato plants between 1 and 21 dpi. Using ANOVA and Tukey’s HSD test, the mean concentrations of GSH and GSSG were calculated at *p* < 0.05. The statistically significant values are marked by letters above the bars.

These results indicated significantly different changes in the concentrations of reduced (GSH) and oxidized (GSSG) glutathione in both potato cultivars. Generally, virus inoculated plants (of both cultivars) showed the induction of GSH content at early stages of infection (1–7 dpi), compared to mock inoculated plants (Figure 3A). In susceptible PVY^NTN^ inoculated Irys plants, the concentration of GSH increased from 1 to 7 dpi (1.31-fold), whereas it significantly decreased (2.84-fold) between 7 and 21 dpi. This suggests that, after 7 days of infection, the susceptible plants could not precisely counteract the oxidative stress. In contrast to susceptible potato plants, the levels of GSH continuously increased from 1 to 21 dpi (1.41-fold) in PVY^NTN^ inoculated hypersensitive Neptun plants. This constant rise in GSH concentration in a resistant cultivar implied that the host precisely regulates and protects cells from oxidative stress. Additionally, the concentration of GSSG changed after PVY^NTN^ inoculation in a different, but linear, manner (Figure 3B). In susceptible PVY^NTN^ inoculated Irys potato, the concentration of GSSG decreased from 1 to 21 dpi (4.62-fold), whereas the PVY^NTN^ inoculated hypersensitive Neptun potato showed a steady increase in GSSG, from 1 to 21 dpi (2.46-fold). This observation indicates that the changes in GSH caused by PVY^NTN^ inoculation allowed the direct change of GSH to GSSG form. Furthermore, the resistant Neptun plants with a higher GSH concentration seemed to use this form in the cell detoxification process and generate more GSSG. Further analyses showed that the modulation of glutathione forms led to significant changes in summary (GSH + GSSG) glutathione content (Figure 3C). In the susceptible Irys potato, the values of summary glutathione content (GSH + GSSG) increased from 1 to 7 dpi (1.20-fold) and significantly decreased after 7 days (2.80-fold) due to PVY^NTN^ inoculation. On the contrary, the summary concentration steadily increased from 1 to 21 dpi (1.54-fold) in PVY^NTN^ inoculated hypersensitive Neptun potato plants. Moreover, analyses showed also that GSSG/GSH + GSSG ratio permanently increased between 1 to 21 dpi in hypersensitive reaction (Figure 4). On the other hand, the GSSG/GSH + GSSG ratio decreased in PVY infected susceptible plants and was lower than in mock inoculated plants. This suggests that, in HR reactions, StGSTs induce the generation of GSSG as a product of the usage of GSH and the generation of GSSG, suggesting a more active protection of the cell in HR reactions.

The increased level of GSSG was highly corelated with changes in *StGSTF2* expression. The analyses of PCC showed that, in PVY infected Irys plants, only at 21 dpi could a positive correlation between GSSG and *StGSTF2* expression level be observed (Table S3A). On the contrary, in the hypersensitive potato Neptun, we observed increased positive correlation between *StGSTF2* and GSSG content, from 3 to 21 dpi (Table S3B). Therefore, the increased relative expression of *StGSTF2* potentially led to more StGSTF2 protein, which catalyzed the generation of GSSG content using GSH.

**Figure 5:**
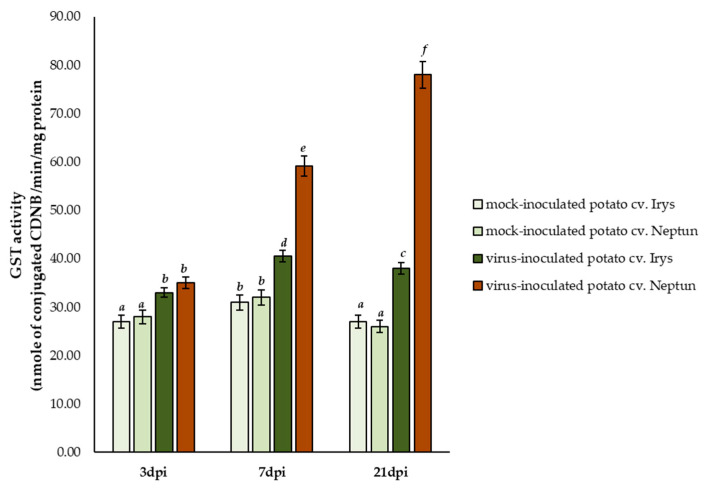
GST activity in *nanomoles* of conjugated CDNB in the leaves of PVY^NTN^ and mock inoculated Irys and Neptun potato plants between 3 and 21 dpi. Using ANOVA and Tukey’s HSD test, the mean concentrations of GSH and GSSG were calculated at *p* < 0.05. The statistically significant values are marked by letters above the bars.

For the in depth analysis of changes in glutathione content in reaction to PVY^NTN^ and the involvement of glutathione in plant host protection against PVY^NTN^ inoculation, GST activity *per se* was also investigated (Figure 5). GST activity can be one of the markers of HR to viral pathogens. The results showed that GST activity significantly increased in PVY^NTN^ inoculated resistant Neptun potato plants, from 3 to 21 dpi (2.23-fold). By contrast, in susceptible infected Irys plants, the activity increased only slightly compared to mock inoculated potato plants, and the increase was observed only between 3 and 7 dpi. At 21 dpi, a decrease in the activity of GST was observed in PVY^NTN^ infected Irys plants. This suggests that a dynamic increase in GST activity can be an indicator of the hypersensitive response of plants to PVY^NTN^, whereas a slight decrease in GST activity can indicate susceptibility.

### 2.4. Subcellular Protection of Cell Organelles by Significant Redistribution Effect of Glutathione (GSH + GSSG) in Resistance to PVY^NTN^

Changes in GSH and GSSG content measured using HPLC and GST activity indicated that the leaf summary glutathione content and glutathione usage were elevated during HR to PVY^NTN^ in Neptun cultivars, especially from 7 dpi. However, this result did not confirm the subcellular redistribution of glutathione, which could be crucial in the reaction to PVY^NTN^ inoculation.

**Figure 6:**
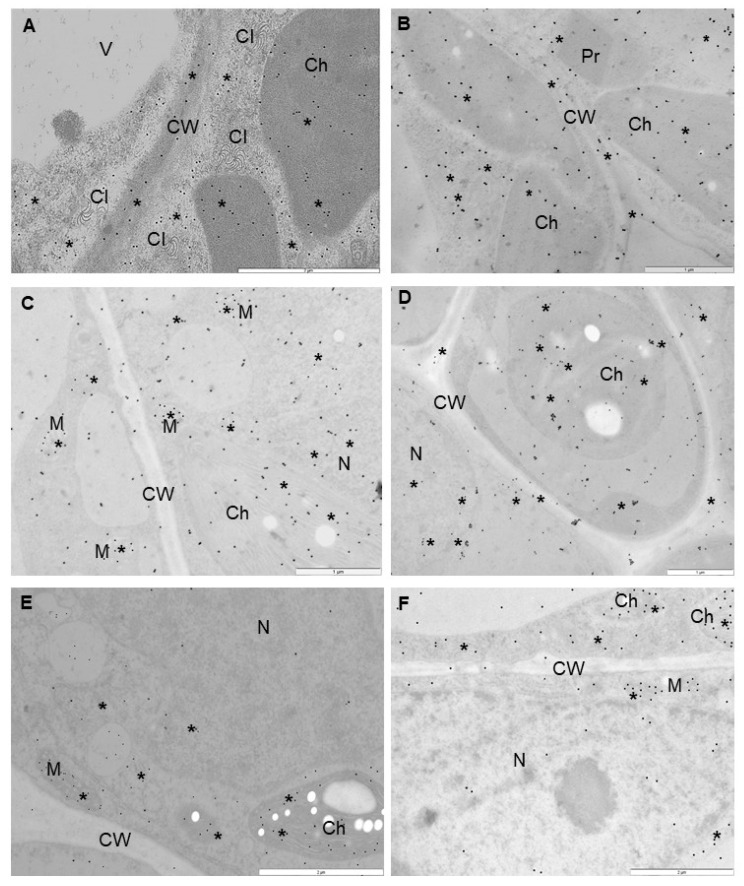
Immunogold labeling of glutathione content in the leaves of PVY and mock inoculated susceptible Irys (**A**,**B**,**E**) and Neptun (**C**,**D**,**F**) potato plants at 7 dpi. (**A**) Glutathione (*) in chloroplast (Ch) and cytoplasm in pallisade mesophyll cell. Virus cytoplasmic inclusions (CI) presented near the cell wall (CW). Bar = 2 μm. (**B**) Glutathione (*) in chloroplast (Ch) and cytoplasm in spongy mesophyll cell. Gold deposition also found in cell wall (CW) and peroxisomes (Pr). Bar = 1 μm. (**C**) Gold granules (*) indicated glutathione in mitochondria (M), chloroplast (Ch), and nucleus (N) in Neptun potato mesophyll cells. Bar = 1 μm. (**D**) Glutathione (*) in chloroplast (Ch) and nucleus (N) in Neptun potato phloem parenchyma cells. Gold granules also present in cell wall. Bar 1 = μm.(**E**) Glutathione localization in chloroplast (Ch), mitochondria (M), and cytoplasm in phloem of potato Irys mock inoculated cells. Bar = 2 μm. (**F**) Glutathione localization in chloroplast (Ch), mitochondria (M), and cytoplasm in the phloem of potato Neptun mock inoculated cells. Bar = 2 μm.

**Figure 7:**
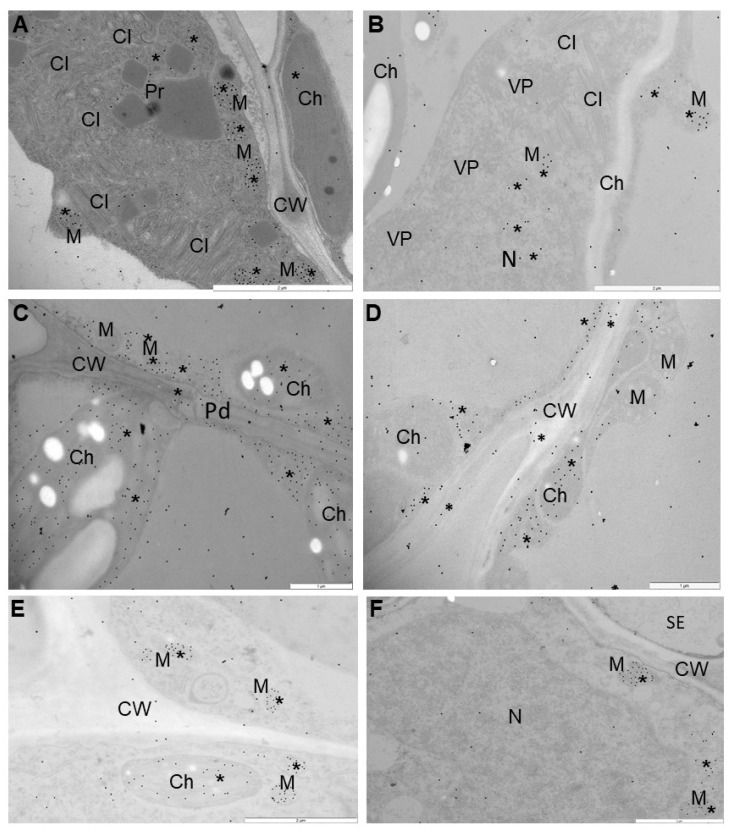
Immunogold labeling of glutathione content in the leaves of PVY and mock inoculated susceptible Irys (**A**,**B**,**E**) and Neptun (**C**,**D**,**F**) plants at 21 dpi. (**A**) Glutathione (*) in mitochondria in palisade mesophyll cells. Virus cytoplasmic inclusion (CI) present in cytoplasm. Bar = 2 μm. (**B**) Gold granules (*) indicated glutathione in mitochondria (M) and cytoplasm. Virus particles (VP) and cytoplasmic inclusion present in cytoplasm of spongy mesophyll cell. Bar = 2 μm. (**C**) Glutathione localization in cytoplasm, chloroplast (Ch), and mitochondria (M) in mesophyll cells. A few gold granules in cell wall around plasmodesmata (Pd). Bar = 1 μm. (**D**) Glutathione localization (*) in cytoplasm and chloroplast (Ch) in phloem parenchyma cells. A few gold granules in cell wall. Bar = 1 μm. (**E**) Glutathione localization (*) in mitochondria (M) and chloroplast (Ch) in phloem parenchyma cells of mock inoculated Irys potato. Bar = 2 μm. (**F**) Weak glutathione localization (*) in nucleus (N) and mitochondria (M) in phloem parenchyma cells of mock inoculated Neptun potato. Bar = 2 μm.

**Figure 8:**
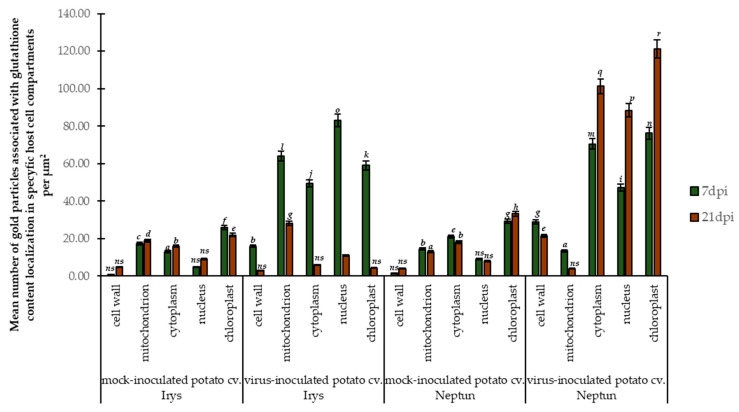
Quantification of immunogold labeling of glutathione content in mock and PVY inoculated Irys and Neptun potato leaves. The figure presents the mean number of gold particles localized in specific compartments per µm^2^ at 7 and 21 dpi in mock and virus inoculated leaves. Statistical validation of immunogold localization was performed using ANOVA. The mean values were calculated at *p* < 0.05 with post hoc Tukey’s HSD test. Statistically significant values are marked with letters above the bars. Nonsignificant values are marked as *ns*.

Therefore, we performed immunogold localization (Figure 6A–F and Figure 7A–F) with proper validation (Figure 8) to determine the exact localization of glutathione content in infected cells. Based on the expression of *StGSTF* genes and changes in glutathione content showed by these analyses, we selected 7 and 21 dpi to present the differences between reactions to PVY infection. Glutathione deposition was induced after virus inoculation in susceptible as well as resistant potato, but the deposition was lower in susceptible potato tissues. Moreover, analysis of localization in the mesophyll and vascular tissues of the susceptible Irys potato indicated that localization in nucleus, mitochondrion, and chloroplast at 7 dpi was higher, in comparison to mock inoculated plants (Figure 6A,B,E and Figure 8). Virus infection was also accompanied by the formation of virus cytoplasmic inclusions (Figure 6A), whereas, in the resistant Neptun potato, the induction in chloroplast, cytoplasm, and nucleus at 7 dpi was more intense in comparison to mock inoculated plants (Figure 6C,D,F and Figure 8). Moreover, in the resistant Neptun potato, the induction of glutathione deposition in the cell wall at 7 dpi was more intense than in susceptible Irys and mock inoculated tissues (Figure 6D,E and Figure 8). In the resistant Neptun potato, glutathione localization in mitochondrion at 7 dpi was at a similar level to mock inoculated tissues. Interestingly, glutathione distribution significantly changed in both susceptible and resistance potato plants at 21 dpi. In Irys plants, PVY infection was fully developed, causing an extreme decrease in glutathione localization. Virus particles and cytoplasmic inclusions were observed in mesophyll and vascular tissues (Figure 7A,B). The level of glutathione was statistically significant only, respectively, in the mitochondrion and nucleus at 21 dpi (Figure 7A,B and Figure 8). This suggested that, at further stages of infection, the susceptible cultivar could not precisely redistribute glutathione and protect the crucial cell compartments from oxidative stress.

On the contrary, glutathione deposition was upregulated compared to the control and even compared with that observed at 7 dpi. Different localization patterns were noticed in the resistant Neptun potato than in the susceptible Irys potato at 21 dpi (Figure 7C–F and Figure 8). In the resistant Neptun potato, localization was most frequently noticed in the chloroplast, cytoplasm, and nucleus, and the most dynamic increase in glutathione deposition was noticed in the chloroplast. On the other hand, a decrease in localization was observed at 21 dpi only in the mitochondria and cell wall in the resistant Neptun potato. This also indicated that the upregulation of glutathione in some regions of the cell results in precise antioxidative protection as needed.

## 3. Discussion

Our study presents, for the first time, the influence of glutathione content and cellular distributions on the susceptible as well as resistant potato–PVY^NTN^ pathosystem. A significant difference in the total glutathione content was observed between susceptible and resistant responses to PVY^NTN^ from 1 to 21 dpi. The highest total glutathione content was noticed in the response of the resistant Neptun potato, which was accompanied by a reduction in virus content from 7 to 21 dpi. The induction of glutathione in the resistant plant can also be related to the induction of defense genes and proteins [37]. Consistent with this statement, the activation of the PR-1 protein, along with a decrease in virus content, was observed with resistance response in the *Turnip mosaic virus*–*Arabidopsis thaliana* respiratory burst oxidase homologs D and F mutant pathosystem [38]. Additionally, our previous study showed the induction of the HRGP-*StExt4* extensin gene during the hypersensitive responses of resistant potato plants to PVY [39], as it is observed in the current study, this is accompanied by the induction of glutathione content. Our analyses indicated that the level of glutathione was lower in the susceptible Irys potato, with the lowest level noted at 21 dpi. In resistance reactions, the glutathione content steadily increased between 1 and 21 dpi, whereas in susceptible responses after virus inoculation, the total GSH content slightly increased up to 7 dpi. Furthermore, in the Irys potato, the glutathione content decreased after 7 days to the lower level compared to mock inoculated leaves. A similar trend was observed by Singh et al. [40] in susceptible and resistance cultivars of *Vigna mungo* inoculated with *Yellow mosaic virus* (YMV) belonging to Begamovirus. The decrease in GSH content between 7 and 21 dpi in the susceptible Irys potato was accompanied by a dynamic increase in virus concentration, and reduced GSH content can be considered as responsible for the development of pathogen elicited symptoms in the susceptible plant, taking into account the conclusions presented by Hernandez et al. [41]. This tendency has been postulated for different susceptible plant–virus interactions; for example, Hakmaoui et al. [42] reported a decrease in ascorbate and GSH levels in *Nicotiana benthamiana* in compatible interaction with the most virulent *Pepper mild mottle virus*. In compatible interaction, such as the PVY^NTN^–Irys potato interaction, when q virus spreads systemically, the main function of glutathione is to protect against oxidative damage by keeping ROS under control; however, it can also lead to plant death. Glutathione and oxidative enzymes also fail to efficiently detoxify ROS in susceptible interactions and to prevent the development of pathogen induced systemic symptoms.

Several studies on different viruses have shown that elevated glutathione improves disease resistance. Gullner et al. [43] showed that the exposure of *Nicotiana tabacum* leaf disc to the cysteine precursor L-2-oxo-thazidine-carboxylic acid, known as OTC, resulted in the accumulation of glutathione and a reduction in TMV content. Similarly, sulfur treatment inhibited the development of symptoms and limited virus content in ZYMV infected pumpkin due to an artificial increase in glutathione [44,45]. Moreover, Király et al. [46] indicated that TMV resistant tobacco with sufficient sulfate showed fewer necrotic symptoms compared to tobacco with a sulfate deficiency. In this experiment, virus resistance correlated with an elevated content of glutathione and Cys and the induction of glutathione.

GSH is necessary for efficient detoxification in plant cells. A significant difference in GSH was observed between PVY^NTN^ resistant and PVY^NTN^ susceptible potato. PVY inoculated potato plants showed induced GSH concentrations compared to mock inoculated potato plants. The dynamic increase in GSH was noticed between 1 and 21 dpi in PVY^NTN^–resistant Neptun potato interactions, whereas, in susceptible potato, GSH increased between 1 and 7 days after virus inoculation and significantly decreased between 7 and 21 days. These data suggest that, in susceptible interactions starting from 7 days, when the first symptoms of PVY^NTN^ inoculation appeared, the Irys potato plant could not counteract oxidative stress. On the other hand, GSH content increased steadily in the resistant Neptun potato, which indicates the regulation and protection of cells from oxidative stress.

Numerous studies have shown that stress conditions have often caused changes in glutathione content and shifted the ratio of glutathione toward the oxidized form [18,47]. In PVY^NTN^–potato interactions, virus inoculation induced the oxidized form of glutathione GSSG compared to mock inoculated plants in both interactions at 1 dpi. However, starting from 3 dpi, the difference in GSSG concentration significantly changed and began to increase. The GSSG concentration decreased in susceptible interactions, whereas a dynamic increase was observed in hypersensitive responses. This observation at 21 dpi was consistent with the data described by Singh et al. [40], who showed that the oxidized glutathione content was the highest in resistant cultivars, in contrast to susceptible cultivars, where it was the lowest. On the other hand, the GSH/GSSG ratio in hypersensitive Neptun potato–PVY^NTN^ interactions was quite different from TMV–tobacco Xanthi in the study by Fodor et al. [23]. Resistant Xanthi showed the elevation in GSH and slightly decreased levels of GSSG in leaves after TMV inoculation. As reported by Király et al. [48] and Künstler [49], a high GSSG level indicated the importance of glutathione in the restoration of TMV resistance, which suggests the suppression of oxidative stress HR in virus infected cells and downstream defense response. In PVY^NTN^–potato interactions, glutathione content increased and the ratio shifted toward GSSG in resistance response, whereas, in susceptible plant–virus interactions, glutathione content decreased compared to resistance response. A similar trend of PVY^NTN^–resistant potato response was observed in the study by Mateo et al. [50], in which an injection of salicylic acid into *A. thaliana* leaves caused an increase in the levels of both GSH and GSSG.

Most of the studies have focused on glutathione content in a whole plant or seedling, whereas glutathione metabolism is cell compartment specific. Moreover, glutathione distribution was found to change during stress conditions. Subcellular changes in glutathione concentration can act as a marker of cellular stress. Therefore, we analyzed, for the first time, glutathione distribution in PVY^NTN^–susceptible potato and PVY^NTN^–resistant potato interaction. Ultrastructural analyses of glutathione content revealed that leaves inoculated with PVY^NTN^ showed significant changes in localization compared to mock inoculated leaves. In susceptible PVY^NTN^ interaction with the Irys potato at 7 dpi, the highest induction of glutathione content was observed in the nucleus, mitochondria, and chloroplast. A similar susceptible interaction with TMV was presented by Höller et al. [51] and Király et al. [46] who showed that glutathione was the most deposited in the chloroplast, nucleus, and mitochondria. After 21 days of PVY^NTN^ inoculation, glutathione content drastically decreased in all compartments, but the highest deposition was still noted in the mitochondria in susceptible responses, which is consistent with the data presented by Zechmann [52] for TMV inoculation at 14 dpi. On the contrary, in hypersensitive responses, the mitochondrion level of glutathione remained unchanged compared to mock inoculated potato leaves after 7 days, when hypersensitivity symptoms appeared. However, between 7 and 21 dpi, glutathione content reduced. These observations are in line with the data reported by Király et al. [46], who indicated that, in incompatible TMV tobacco infection glutathione depletion induced in the mitochondria correlated with the induction of necrotic lesions in hypersensitive responses. A similar trend was observed in *B. cinerea* interaction by Simon et al. [53]. It can be postulated that, in incompatible PVY^NTN^–interactions, the glutathione level in mitochondria decreases and also changes toward the GSSG form. This can lead to mitochondrial dysfunction as well as the activation of plant defense responses and resistance. It has been shown that, during compatible interaction, glutathione levels increased or were simulated in symptoms, but not cell death [54]. This suggests that the primary function of glutathione in mitochondria is to keep ROS under control and, thus, save cells from damage and cell death.

Another important organelle for ROS generation and defense signaling is chloroplast [2]. In PVY^NTN^–potato interactions, glutathione induction in the chloroplast occurred at 7 dpi in susceptible reactions as well as in HR. However, between 7 and 21 dpi, the level of glutathione levels significantly decreased in susceptible reactions. As reported by Noctor et al. [55], the decline in GSH in the chloroplast, despite active synthesis in this organelle, may be related to its transport to other cell compartments. In contrast to susceptible glutathione reduction after PVY^NTN^ inoculation, in HR the level of glutathione increased more dynamically and elevated to the highest value from all other cell compartments at 21 dpi. Our observations reflect that of Höller et al. [51] and Zechmann [52] in TMV susceptible and TMV resistant tobacco interactions. As postulated by Clemente-Moreno et al. [56], ROS accumulation is a common feature in potyviral infection (*Plum pox virus*, PPV). Our observations related to PVY^NTN^ interaction reflect the data on PPV and TMV infection, which showed that, at 7 dpi, susceptible plants revealed the strongest increase in glutathione content in the chloroplast. Elevated glutathione concentration in the chloroplast is also an important factor for ROS control and the development of symptoms. The breakdown of the oxidative system in the chloroplast can be correlated with necrosis. Taken together, it can be concluded that ROS is controlled by glutathione in the chloroplast and under control systemic symptoms, death occurs.

In PVY^NTN^–Irys potato interaction, the highest localization of glutathione content was observed in the nucleus at 7 dpi, whereas in hypersensitive responses the most intense glutathione induction in the chloroplast and cytoplasm occurred between 7 and 21 dpi. In TMV infected tobacco and *Arabidopsis* inoculated with *P. syringae* or *B. cinerea*, Király et al. [46] and Simon et al. [53], respectively, demonstrated that the induction of glutathione in the nucleus was followed by a strong accumulation in the chloroplast as well as in the cytoplasm. Moreover, in many plant–pathogen interactions, the increase in glutathione in the nucleus indicates the elevation of total glutathione, while glutathione can diffuse into the nucleus after synthesis in the cytoplasm [57,58]. In general, the accumulation of glutathione in the nucleus indirectly leads to plant defense by providing a reducing environment for antioxidant enzymes involved in transcription and protein modification [59]. Glutathione localization in PVY^NTN^ resistant Neptun potato plants steadily increased, whereas in susceptible interactions with the Irys potato a dynamic decrease was noticed in the later stages of infection, at 21 dpi. Based on the results observed in *Arabidopsis* mutant, it was postulated that a lower level of glutathione enhanced susceptibility to pathogens, which highlighted that a sufficient supply of glutathione is very important for efficient plant defense [60]. At an adequate level, glutathione counteracts ROS as well as activating defense related genes and the accumulation of related proteins, such as pathogenesis related protein group (PR protein). Cytoplasm also plays an essential role in glutathione metabolism during defense.

Significant differences in glutathione deposition were also noticed in the cell wall in PVY^NTN^–potato plant interactions. In the cell wall, glutathione localization was induced after 7 days of PVY^NTN^ inoculation and the process was more dynamic in hypersensitive responses than in susceptible reactions. The cell wall glutathione content decreased between 7 and 21 dpi in both reaction types, but in susceptible interaction the decrease in content was insignificant. Furthermore, the increase in the cell wall glutathione pool in HR corresponds with the induction of hydroxyproline rich glycoprotein and HRGP-extensin genes and the localization of extensins, which is line with the cell wall reinforced PVY^NTN^–potato hypersensitive reaction observed in our previous work [39]. According to Tolin et al. [61], GSH content and redox state in the apoplast are of importance in sensing and signaling stress. The apoplast glutathione pool becomes more oxidized during hypersensitive responses and plays a key role in adaptation to biotic stress [62].

Plant GSTs (EC 2.5.1.18) are essential enzymes involved in diverse functions, such as detoxification, redox homeostasis, plant metabolism, signaling, and especially regulation and adaptation to abiotic and biotic stress [25]. Transcriptome profiling of pepper leaves in compatible and incompatible pepper tobamovirus interactions revealed that infection by *Obuda pepper virus* (ObPV) strongly induced GST genes [63]. The inoculation of ObPV resulted in the activation of cysteine and GSH biosynthesis pathway. Kalapos et al. [63] indicated that 22 GSTs were highly induced at 3 dpi. Genome wide analyses of GST genes have been performed in various plants, and the results revealed the presence of 55 genes in *Arabidopsis* [64] and a total of 90 genes in *S. tuberosum* [32]. The results also highlighted that potato GST proteins could be divided into 10 major classes, of which the two largest are tau (66 members) and phi (5 members). Furthermore, expression profiling indicated that all phi members were highly expressed in leaves, and the analysis of *StGST* phi genes revealed that *StGSTF* genes are expressed as a response to biotic stress [32]. Therefore, we decided to estimate the expression of selected *StGSTF* genes in the PVY^NTN^ resistant Neptun potato as well as in the susceptible Irys potato. Normalized relative expression analyses clearly indicated the differential expression of *StGSTF1*, *StGSTF2*, and *StGSTF5* during hypersensitive and susceptible reactions between 3 and 21 days after PVY^NTN^ inoculation. *StGSTF1* showed induced expression in both interaction types compared to mock inoculated plants at 3–21 dpi. It could be observed that the expression profile did not significantly differ between the resistant and susceptible potatoes, whereas *StGSTF2* was highly induced, especially in HR, compared to mock inoculated Neptun plants. On the contrary, in the susceptible response of the Irys potato, the expression of *StGSTF2* increased between 3 and 7 dpi, but significantly decreased between 7 and 21 dpi. Significant differences were observed in the expression profiles of *StGSTF1* and *StGSTF2* genes compared to mock inoculated potato plants, whereas the expression of *StGSTF5* seems to be unchanged compared to the mock inoculated Irys and Neptun potato plants. As underlined by Islam and co-authors [32], *StGSTF2*, analyzed by us, belongs to the highly expressed glutathione transferases genes in potato tissues. Whereas *StGSTF1* does to the medium level of expression. Furthermore, according Islam and co-authors [32], *StGSTF2* and *StGSTF5* were highly expressed in leaves, with *StGSTF1* on the lower level. On the other hand, *StGSTF1* and *StGSTF2* were postulated as highly induced in pathogen response, while *StGSTF5* as a bit weaker than *StGSTF2*. Moreover, strongly positive correlation from 3 to 7 dpi between *StGSTF2* and *PVY-CP* genes was confirmed in susceptible reactions. Whereas, after 7 dpi, the correlation became negative and a strong decreased expression of *StGSTF2* was correlated with an increase in *PVY-CP* relative expression level. In hypersensitive responses, the upregulated expression of *StGSTF2* was corelated with the down regulation of *PVY-CP.* Our observations reflect that, in PVY^NTN^–potato plant interactions, *StGSTF2* is the most affected, and its expression differs between susceptible and resistant reactions and acts as a marker of response to PVY^NTN^. The decrease in GSH content between 7 and 21 dpi in susceptible reactions was accompanied also by a dynamic increase in PVY^NTN^ concentration, therefore, we supposed that it can be responsible for the development of virus elicited symptoms in susceptible plants. StGSTF2, along with glutathione control, may contribute to susceptibility by supporting viral replication. In another situation this was observed in hypersensitive responses, where GSH and GSSG contents increased, correlating with an increase in *StGSTF2* expression. Therefore, the positive correlation between “components” were confirmed from 3 to 21 dpi. StGSTF2 used GSH in potato plant cells and “transform” to the GSSG form to save or rescue the cell from damaging oxidative stress.

These changes in gene expression were accompanied by GST activity in both potato cultivars inoculated with PVY^NTN^. However, in hypersensitive responses, the steady induction of GST activity was noticed between 3 and 21 days after PVY^NTN^ inoculation, but the most dynamic induction was found between 7 and 21 dpi. On the contrary, in the susceptible potato, a slight increase in GST activity was observed between 3 and 7 days after PVY^NTN^ inoculation followed by a decrease after the first symptoms appeared, between 7 and 21 dpi. Previous analyses of antioxidative genes in susceptible cultivars Igor and Nadine inoculated with aggressive and mild PVY revealed that the expression of genes was lower in cultivars inoculated with aggressive PVY than those inoculated with mild PVY [65]. The most pronounced difference between cultivars was the variation in the expression of GST at 2 days after virus inoculation in susceptible reactions. GST may have a pivotal function in controlling HR and necrotization during plant–virus interaction, as was postulated by Fodor et al. [23]. Additionally, the increased expression of *NtGSTU1* (from the *tau* group) was observed between 3 and 6 h after virus inoculation, which manifested as enhanced HR, causing a reduction in TMV replication in plants with sufficient sulfate [46]. Similar to PVY^NTN^–Neptun potato HR, the enhanced expression of GST genes correlated with HR induction and reduced virus levels, according to Ishihara et al. [66]. Brizard et al. [67] stated that GST was purified during infection by *Rice yellow mottle virus* in a partially resistant cultivar, but not in a susceptible cultivar. Expression of GST genes was significantly induced only in the beet necrotic yellow vein virus resistant line [68]. It was also shown that GST activity promoted resistance to the virus [69]. More than 50% of the increase in GST activity was noted in the resistant sorghum cultivar in the first 3 days after inoculation with sugarcane mosaic virus, whereas susceptible cultivars showed a strong decrease in GST activity [69]. Therefore, it was assumed that slightly increased or decreased GST activity may lead to only weak resistance or even susceptibility. In the PVY^NTN^–potato pathosystem, the relative expression of *StGSTF2* reflects a steady increase in hypersensitive response, in the contrary to susceptible response the increase up to 7 dpi was observed and between 7 and 21 dpi decrease. A similar tendency was observed in GST activity, but taking into account *StGSTF1* expression levels, the tendence differed. In relative expression analyses, we checked three GST genes belonging to the one *phi* group of potato GSTs. Whereas GSTs activity was analyzed based the whole pool of glutathione transferases in potato. Therefore, the activity and gene expression data can be different. Further analyses, also on other glutathione transferases, will be needed and it is very important to shed new light on the correlation between the expression of different transferases groups and tendencies in StGSTs activity. On the other hand, several GST genes were induced in susceptible RTSV interaction, but no visible systemic symptoms appeared [70]. On the contrary, in *A. thaliana* susceptible to cauliflower mosaic virus (CaMV), compatible interactions caused the systemic induction of GST1, which was accompanied by increased CaMV levels and systemic mosaic symptoms [71]. However, Chen et al. [72] showed that *NbGSTU4* was upregulated in *Bamboo mosaic virus* in *N. benthamiana*. *NbGSTU4* binds to (+) RNA in a GSH dependent manner and is even essential for efficient virus replication.

Summarizing, plant GSTs may be involved in the establishment of resistance to virus infections, with or without oxidative stress; however, they could also contribute to limiting oxidative stress during susceptibility and systemic infection. GST, along with glutathione control, contributes to virus susceptibility by supporting viral replication. A comparison between compatible and incompatible reactions indicated that GSTs can play an important role in disease resistance, but the underlying molecular mechanism is still unclear. Further studies are needed with overexpressing and/or reduced lines of individual GSTs for a more in depth understanding of the resistance mechanism.

## 4. Materials and Methods

### 4.1. Plant Material, Virus Inoculation, DAS-ELISA and PVY Concentration

During investigation, potato plants (*S. tuberosum* L.) of two cultivars—susceptible Irys (resistance level of 5.5 on a scale of 1–9) [33] and resistant Neptun (resistance level of 8 with confirmed hypersensitive reaction (HR) [33,34,35]), which were obtained from IHAR-PIB, Plant Breeding and Acclimatization Institute, Bonin Research Center, were inoculated with the NTN strain of PVY (PVY^NTN^). The Neptun cultivar exhibits HR to infection by PVY^NTN^ and consists of the marker S1d11, the presence of which is associated with that of resistance gene Ny-1 on chromosome IX, a finding similar to that revealed by Rywal [73]. The cultivation and PVY^NTN^ inoculation of potato seedlings (at the four leaf stage) were performed according to previously described procedures [39,74]. The Neptun cultivar showed a hypersensitive necrotic response visible at 6–7 dpi (Figure S1), while the Irys cultivar showed systemic necrosis at 10–15 dpi (Figure S1). The leaves of mock and PVY^NTN^ inoculated plants were evaluated for the presence of the virus by the double antibody sandwich enzyme linked inmunosorbent assay (DAS-ELISA) technique, as described by Kozieł et al. [75], using primary monoclonal antibodies against PVY^NTN^ (Bioreba, Reinach, Switzerland) and purified secondary antirabbit antibodies conjugated with alkaline phosphatase (Bioreba, Reinach, Switzerland) [76]. Each repetition of the experiment was performed on a new ELISA plate. Each test was performed on samples collected from 30 mock inoculated plants (both cultivars) and combined separately, and the same protocol was applied for 30 PVY^NTN^ inoculated plants (both cultivars). All DAS-ELISA tests were performed using the same reagents. The OD_405nm_ values were read after 60 min in duplicates at 3, 7, and 21 dpi, and the mean values were statistically analyzed by analysis of variance (ANOVA), as described by Kozieł et al. [75], using Statistica software (version 13.0; StatSoft and TIBCO Software Inc., Palo Alto, CA, USA). Furthermore, the OD_405nm_ values were validated by estimating the corrected mean OD_405nm_ values, as previously described [75], and used for a precise comparison of the relative levels of virus presence/concentration in plants. The cut off point was calculated using a formula suggested by Bioreba (Switzerland) [77] and Otulak-Kozieł et al. [38] and found to be 0.129. The readings at OD_405nm_ were compared to the calculated cut off point, and all OD_405nm_ values that were greater than 0.129 were considered positive (i.e., confirmed the presence of the virus) [38]. The significant threshold/cut off point values obtained after DAS-ELISA confirmed the presence of virus in all inoculated potato plants. To double check level of PVY we performed qPCR of *PVY-CP* gene fragment with use of primers presented by Abdalla et al. [78]: 5′-GATGGTTGCCTTGGATGATG-3′ (forward primer) and -5′-TAAAAGTAGTACAGGAAAAGCCA-3′ (reverse primer) in comparison to mean expression of the plant host reference genes *StEf1α* (*S. tuberosum* elongation factor-1 alpha) and *Stsec3* (exocyst complex component). The level of the virus is presented in a form of normalized expression of *PVY-CP* gene. For DAS-ELISA, *PVY-CP* expression and also other performed analyses (microscopy, HPLC, GST enzymatic activity), we use 60 plants (30 virus inoculated and 30 mock inoculated of each potato cultivar). The analyses were performed in 3 repetitions each time on the new group of 60 plants.

### 4.2. Isolation of RNA and Genomic DNA (gDNA) for GST Gene in PVY^NTN^-Infected Potato Plants

To estimate the expression of glutathione S-transferase phi (GSTF) genes in plant host, molecular analyses were performed on the samples collected at the same time intervals as those used in the microscopic studies of glutathione content localization. Briefly, leaf samples (0.1 g of each sample) were collected from 30 mock (buffer) or virus infected seedlings per cultivar at 3, 7, and 21 dpi. RNA isolation, purification, and quality analyses were carried out as described previously [39,79]. Additionally, the absence of RNA contamination was verified by performing reverse transcription-polymerase chain reaction using *StEf1α* (*S. tuberosum* elongation factor-1 alpha) and *Stsec3* (exocyst complex component) as the reference standard [39,80], which confirmed the absence of contaminating gDNA. Thereafter, cDNA was synthesized using the NG dART RT kit (EURx Sp. z o.o., Gdansk, Poland), as per the recommended protocol. Reverse transcription reactions were performed in a 10-µL volume using 1000 ng of RNA.

### 4.3. Analysis of Expression of Selected GSTF Genes in PVY^NTN^-Infected Potato Plants Using qPCR

A real time quantitative polymerase chain reaction (qPCR) was performed using the Bio-Rad CFX96TouchTM apparatus (Bio-Rad Poland Sp. z o.o., Warsaw, Poland) and Fast SG qPCR Master Mix (2x) (EURx Sp. z o.o., Gdansk, Poland) for StEf1α reference gene. All qPCR tests were calibrated with previously prepared five point calibration curves (based on cDNA and gDNA). The following genes were analyzed in qPCR: *S. tuberosum GSTF1* (*StGSTF1*, Sotub02g024450.1.1), 2 (*StGSTF2*, GeneID: XM_006355737.2, Sotub06g007440.1.1), and 5 (*StGSTF5*, Sotub12g027670.1.1) [32]. These host genes encoded protein products that were associated, respectively, with the utilization of glutathione in response to biotic and abiotic stress (GST). Moreover, Islam et al. [32] showed that *StGSTF1, StGSTF2, and StGSTF5* genes are mainly related to potato leaves and their expression is modified in potato during pathogen infection. The analyses of these authors in CELLO and pSORT programs revealed the involvement of the product of *StGSTF1, StGSTF2, and StGSTF5* genes in chloroplast and cytoplasm of potato cells and that of StGSTF5 gene in nucleus [32]. The expression of *StGSTs* in *S. tuberosum* was analyzed, and complete sequences were determined and published in SpudDB Potato Genomic Resource [81]. Gene expression was investigated in both types of potato cultivars using *S. tuberosum StEf1α* (GeneID: AB061263) and *Stsec3* reference genes (PGSC0003DMG402015451), as previously described [39,80]. The primers were designed using Primer3 software (version 0.4.0; Primer3Plus, Free Software Foundation, Inc., Boston, MA, USA). Table S2 presents all the primers used in the experiments. The starting cDNA solution (used for generating calibration curves) was a fourfold diluted mix of 12 randomly selected cDNA mixes. An eightfold diluted cDNA mix was used for constructing the calibration curve for gDNA. The subsequent calibration points were measured at fourfold dilutions in a 15-µL volume. A 5-µL solution of eightfold diluted cDNA mix was added to the reaction mixture. The conditions used for qPCR analyses are presented in Table S3.

### 4.4. High-Performance Liquid Chromatography (HPLC) Analysis of Reduced (GSH) and Oxidized (GSSG) Forms and Summary Glutathione Content

The content of GSH and GSSG was measured by reversed phase HPLC with fluorescence detection, as described by Kranner [82]. Briefly, leaves (120–180 mg) were ground in liquid nitrogen, and immediately homogenized with 1.8 mL of 0.1 mM HCl containing 1 mM EDTA and polyvinylpolypyrrolidone (with the same amount as plant material). Then, the samples were mixed well, shaken for 20 min, and centrifuged (20,000× *g*, 20 min, 4 °C). From the resulting supernatant, 120 μL was used for the determination of total glutathione content and 400 μL for estimating the content of GSSG. For the determination of total glutathione content, 120 μL of the supernatant was mixed with 180 μL of 200 mM CHES buffer (pH 9.3, to adjust pH to 8.0–8.3) and 30 μL of 3 mM dithiothreitol (DTT). The samples were incubated at room temperature for 60 min. Then, 20 μL of 15 mM monobromobimane (MBBr; Sigma, 69898, Warsaw, Poland) was added to begin derivatization, which was carried out for 15 min at room temperature in darkness. Next, the samples were acidified using 20% acetic acid, centrifuged (13,000× *g*, 5 min, 4 °C), and 10 μL of the solution was injected into the HPLC system. For validating the content of GSSG, 400 μL of the extract was incubated with 30 μL of 50 mM N-ethylmaleimide (NEM) and 600 μL of 200 mM CHES buffer for 15 min at room temperature. To remove the excess NEM, the extract was mixed with the same volume of toluene and vortexed for 30 s. Once the phases were separated, the toluene phase was discarded. This step was repeated 6 times. Next, 30 μL of 3 mM DTT was added to 300 μL of the sample and the mixture was incubated for 60 min. The samples were derivatized as described above. Bromine derivatives were chromatographically separated using Bionacom Velocity C18 LPH (4.6 × 150; 3 μm) column at 35 °C. The peaks were detected using FP-2020/2025 Intelligent Fluorescence Detector (JASCO) (Ex 390 nm; Em 478 nm). The following was used as a mobile phase: 0.25% acetic acid containing 5% methanol, adjusted to pH 3.9 with 5 M NaOH (A) and 100% methanol (B). The flow rate of eluents was maintained at 1 mL min^−1^. The gradient program was as follows: 0–5 min, 80–75% (A); 5–30 min, 75–70% (A); 30–38 min, 70–0% (A); and 38–45 min, 0–80% (A). Measurements were carried out in three biological replicates, each in 2 technical replicates. The content of GSH and GSSG was estimated using the results of standards and presented as μg g^−1^ FW (fresh weight). Moreover, based on HPLC results we analyzed further the ratios: GSSG/GSH + GSSG content to check more precisely generation of this glutathione form.

### 4.5. Validation of GSTs Activity in Leaves of PVY^NTN^-Infected Potato Plants

Potato leaves were collected at 3, 7, and 21 dpi after the inoculation of mock or PVY. The total protein content was extracted using ice cold extraction buffer containing 100 mM potassium phosphate buffer (pH 7.0), 50% glycerol, 16 mM MgSO_4_, and 1 mM PMSF57, as described by Islam et al. [83]. After the quantification of protein using the Bradford method [84], the activity of GST was determined based on its ability to conjugate GSH and 1-chloro-2,4-dinitrobenzene (CDNB) at 344 nm [85]. The determination of activity was performed using the extinction coefficient of the product formed (9.6 mM^−1^ cm^−1^), and the result was expressed as nanomoles of CDNB conjugated/min/mg total protein.

### 4.6. Immunogold Localization of Glutathione Content Changes in PVY^NTN^-Infected Potato Plants

Based on the analyses of virus concentration for microscopic studies, the leaf samples of (mock and virus inoculated) potato plants at 7 and 21 dpi were embedded and treated as described by Zechmann et al. [57] and Kolb et al. [86]. Then, the leaf sections were mounted on Formvar coated nickel grids, and immunogold localization was carried out as described by Zechmann et al. [57]. The sections were counterstained with 2% uranyl acetate for 5 min and washed 5 times for 2 min each with distilled water. For determining the localization of glutathione content, we used primary polyclonal rabbit antibodies targeted to summary glutathione (in 1:100 dilution; Merck, Warsaw, Poland) and visualizing secondary antirabbit antibodies conjugated with 18 nm nanogold particles (Jackson ImmunoResearch Europe Ltd., Cambridgeshire, UK). The labeling specificity was checked by incubating the grids with the samples obtained from mock inoculated plants and by omitting the primary antibodies from the incubating solution. The immunogold labeled sections on grids were examined using a transmission electron microscope [87]. After examination, protein labeling was quantified using the method of Luschin-Ebengreuth and Zechmann [88]. Statistical analyses were performed as described by Otulak-Kozieł et al. [87]. The concentrations of gold particles in specific cell sections were validated using ANOVA and post hoc Tukey’s HSD test using Statistica software (version 13.0; StatSoft and TIBCO Software Inc., Palo Alto, CA, USA). ANOVA was used to estimate gold labeling. For the statistical estimation of immunogold labeling, infected and mock inoculated materials were compared at 3, 7, and 21 dpi. The number of gold particles in cell compartments was counted in 35 fields (10 μm^2^) per image. For each combination (two mock inoculated plants and PVY^NTN^ inoculated Irys and Neptun potato plants), gold particles from 200 photos were counted to determine the presence of glutathione content.

### 4.7. Pearson’s Correlation Coefficients (PCCs) for Analyses

Based on data from expression of *StGSTF2*, levels of PVY (relative expression of *PVY-CP*) and GSSG content (in PVY infected plants) correlation analyses was performed. To compare/check the pairwise likelihood between *StGSTF2* changes and levels of PVY, as well as likelihood between *StGSTF2* expression and GSSG content, Pearson’s correlation coefficients (PCCs) were estimated according to Wu et al. [89] and Manders et al. [90] by using Excel 2019 software (Microsoft, Poland, Warsaw). The pairwise correlations between *StGSTF2* changes and levels of PVY as well as *StGSTF2* expression and GSSG content were estimated at 3 dpi, 7 dpi, in hypersensitive and susceptible reactions. The results were presented in the form of a heat map generated using PCC values, and values over 0.70 were considered as to reflect the strong positive correlation between analyzed pairs.

## 5. Conclusions

Although several studies have focused on glutathione in plant–virus interactions, the available information is still far from complete. Therefore, we investigated, for the first time, the influence of glutathione content as well as its distribution at an ultrastructural level on susceptible and hypersensitive potato–PVY^NTN^ pathosystems. Our results clearly indicated that PVY^NTN^ inoculation resulted in glutathione induction in both resistance and susceptible response at the first steps of plant–virus interaction, but the induction of hypersensitive responses was much more dynamic and accompanied by a reduction in virus content.

In the resistance reaction, the glutathione content steadily increased between 1 and 21 dpi. We revealed that enhanced expression of *StGSTF2* corresponded with an increase in GSSG content as well as HR induction and reduced PVY^NTN^ concentration. Additionally, ultrastructural distribution indicated that glutathione was mostly deposited in the chloroplast, cytoplasm, and nucleus. Glutathione also plays a very important role in these compartments—it activates plant defense and is involved in the development of resistance. Moreover, it keeps ROS under control and reduces potential damage to the host plant cell.

On the contrary, in susceptible responses, the total glutathione content slightly increased after PVY^NTN^ inoculation, but only up to 7 dpi. Moreover, between 3 and 7 days after PVY^NTN^ inoculation in susceptible responses, an increase in GST activity was observed, followed by a decrease after the first symptoms appeared at the later stages of infection, between 7 and 21 dpi. Furthermore, these tendencies correlated with *StGSTF2* relative gene expression. Ultrastructural distribution indicated that the nucleus, chloroplast, and mitochondria were the compartments where glutathione accumulated the most in PVY^NTN^–Irys potato but the highest localization was observed only up to 7 dpi, and after symptoms appeared glutathione deposition drastically reduced. It was confirmed that *StGSTF2* participates not only in resistance response but also in the limitation of oxidative stress in susceptibility and systemic virus infection. *StGSTF2*, along with glutathione control, contribute to susceptibility by supporting viral replication. Additionally, the decrease in GSH content between 7 and 21 dpi was correlated with a dynamic increase in PVY^NTN^ concentration, which can be responsible for the development of virus elicited symptoms in susceptible plants. A comparison between compatible and incompatible interactions indicated that StGSTs can be involved in disease resistance, but the underlying molecular mechanism is not completely understood. Therefore, further extended research is needed using overexpressing or reduced/silenced lines of individual GST from different GST groups for a more in depth understanding of the resistance mechanism.

## Supplementary Materials

The following supporting information can be downloaded at: https://www.mdpi.com/article/10.3390/ijms23073797/s1.
