# Supplementary material for: Glutathione Modulation in PVYNTN Susceptible and Resistant Potato Plant Interactions
Source: Int J Mol Sci. 2022 Mar 30;23(7):3797. doi: 10.3390/ijms23073797 (PMC8998174; doi:10.3390/ijms23073797)
Supplement: Supplementary file 1 [file ijms-23-03797-s001.zip › ijms-1644222-supplementary.pdf]

Table S1. PVY^{NTN} detection using DAS-ELISA assay in mock- and virus-inoculated potato plants as reflected by mean OD405nm values. The absence of a virus is marked by (−). The presence of PVY^{NTN} (+) is considered positive when the mean OD405nm values over the cut-off point (0.129).

Sample	Mean OD_{450nm}	Presence (+)/Absence of the Virus (−)
Buffer	0.0000	−
Mock-inoculated potato cv. Irys (3 dpi)	*0.0400*	−
Mock-inoculated potato cv. Neptun (3 dpi)	*0.0389*	−
PVY^{NTN}-inoculated potato cv. Irys (3 dpi)	*0.7493*	+
PVY^{NTN}-inoculated potato cv. Neptun (3 dpi)	*0.5932*	+
Mock-inoculated potato cv. Irys (7 dpi)	*0.0408*	−
Mock-inoculated potato cv. Neptun (7 dpi)	*0.0404*	−
PVY^{NTN}-inoculated potato cv. Irys (7 dpi)	*1.0698*	+
PVY^{NTN}-inoculated potato cv. Neptun (7 dpi)	*0.8964*	+
Mock-inoculated potato cv. Irys (21 dpi)	*0.0440*	−
Mock-inoculated potato cv. Neptun (21 dpi)	*0.0611*	−
PVY^{NTN}-inoculated potato cv. Irys (21 dpi)	*3.003*	+
PVY^{NTN}-inoculated potato cv. Neptun (21 dpi)	*0.3001*	+

Supplement Table S1

Table S2. Heatmap of PCC for *StGSTF2* normalized expression (based on *StEf1α*) and PVY levels in virus-inoculated susceptible potato Irys (**A**) and hypersensitive potato Neptun (**B**) from 3 to 21 dpi. PCC matrix values are presented pairwise for specific cell compartments in specific time dpi and marked with colors, from very dark green (PCC = 1) to bright green (PCC = -1) in susceptible reaction and , from very dark brown (PCC = 1) to bright brown (PCC = -1) in hypersensitive reaction.

A

cv. Irys		Normalized expression *StGSTF2*		
		3 dpi	7 dpi	21 dpi
PVY levels	3 dpi			
	7 dpi			
	21 dpi			

-1
0
0.7
1

B

cv. Neptun		Normalized expression *StGSTF2*		
		3 dpi	7dpi	21 dpi
PVY levels	3 dpi			
	7 dpi			
	21 dpi			

-1
0
0.7
1

Supplement Table S2

Table S3. Heatmap of PCC for *StGSTF2* normalized expression (based on *StEf1α*) and GSSG content in PVY-inoculated susceptible potato Irys (**A**) and hypersensitive potato Neptun (**B**) from 3 to 21 dpi. PCC matrix values are presented pairwise for specific cell compartments in specific time dpi and marked with colors, from very dark green (PCC = 1) to bright green (PCC = -1) for susceptible reaction and, from very dark green (PCC = 1) to bright green (PCC = -1) for hypersensitive response.

A

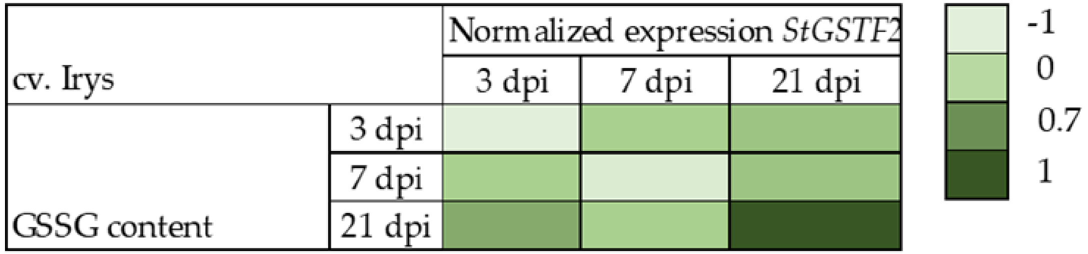

B

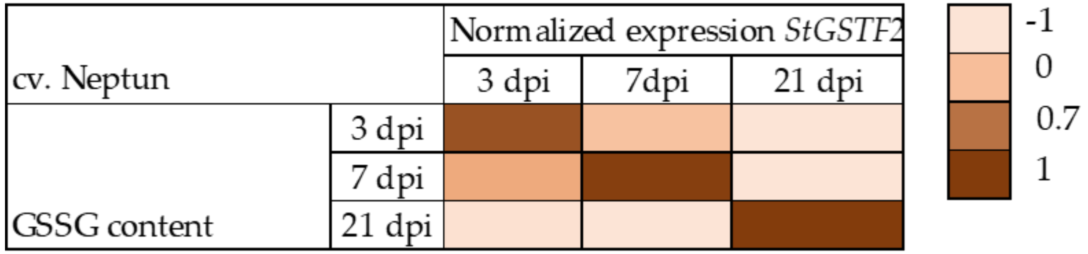

Supplement Table S3

Supplement Figure S1

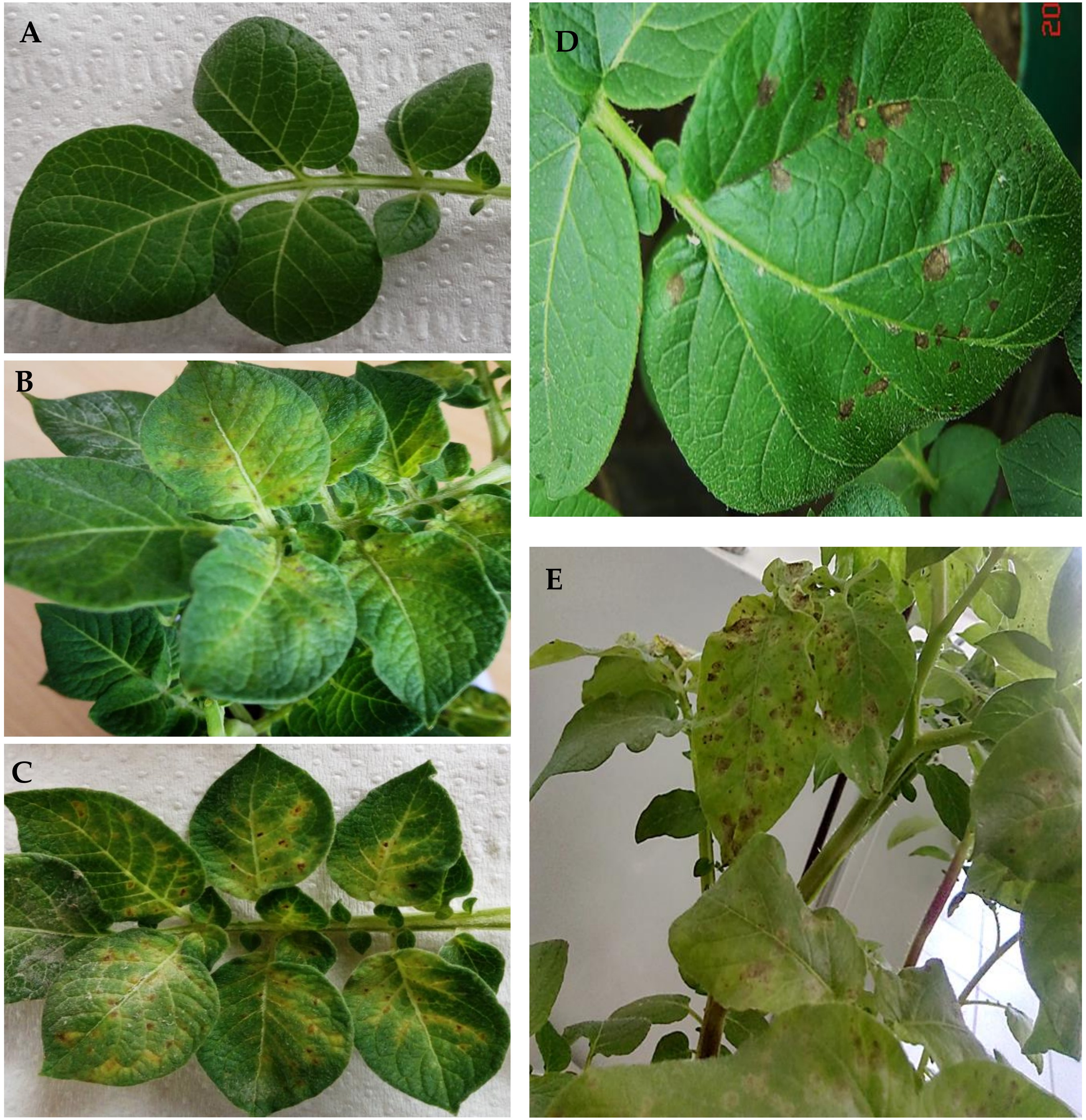

Figure S1. Symptoms of PVY^{NTN} inoculation:
(A) Healthy (mock-inoculated) potato leaflet;
(B) Local symptom on inoculated Neptun leaf 7 dpi
(C) Local symptoms on inoculated Neptun leaf 14 dpi
(D) Irys leaf 7 days after inoculation, local symptoms appeared
(E) Irys plant 21 days after inoculation, systemic symptoms

Table S4 Gene accession numbers, primer sequences, and product lengths for RT-qPCR analyses.

Genes	*Gene ID*	Forward Primer	Reverse Primer	Temp of Primer Annealing (°C)	Concentration in Reaction (µM)	Product Length (bp)
**Investigated**						
StGSTF1	*Sotub02g024450.1.1*	5′-GTGGGGGTAGGGATAAGGAA-3′	5′-CAATGGACTGGGCTGATTTT-3′	58	0,5	243
StGSTF2	*Sotub06g007440.1.1*	5′-GGCAGAGAACGAAGAGAAAC -3′	5′-GGCAGAGAACGAAGAGAAAC -3′	58	0.5	90
StGSTF5	*Sotub12g027670.1.1*	5′-ACTCCGGTGAAAGTGTACGG-3′	5′-GGGGTAGGAGGGAAAATTGA-3′	58	0.5	232
**Reference**						
StEF1a	AB061263	5′-GGTGATGCTGGTATGGTTAAG-3′	3′-GGTCCTTCTTGTCAACATTCTT-5′	58	0.5	148
Stsec3	PGSC0003DMG402015451	5′-GCTTGCACACGCCATATCAAT-3′	3′-TGGATTTTACCACCTTCCGCA-5′	58	0.5	106

Supplement Table S4

Table S5. Conditions of the RT-qPCR for the reference genes (*).

* Fluorescence signal reading was taken at the final stage

Program	Parameters
Preliminary denaturation	95 °C for 5 min
Amplification (35 cycles)	95 °C for 10 s<br>58 °C for 10 s<br>72 °C for 20 s *
Melting curve	65–95 °C; 0.1 °C/s

Supplement Table S5
